# Bridging Employees’ Perceptions of Corporate Social Responsibility, Sense-Making for Meaningfulness, and Work Engagement for Successful Self-Regulation

**DOI:** 10.3390/bs14111014

**Published:** 2024-10-31

**Authors:** Zheni Wang, Steve Carroll, Eric H. Wang

**Affiliations:** 1 Department of Management/International-Business, School of Business, Southern Connecticut State University, New Haven, CT 06515, USA; steveecarroll82@gmail.com; 2 Department of Electrical and Computer Engineering, College of Engineering, University of California, Davis, CA 95616, USA; eriwang@ucdavis.edu

**Keywords:** micro-CSR, work engagement, calling work orientation

## Abstract

In response to calls for research on the psychological mechanisms, such as perceptions and attitudes toward corporate citizenship, in promoting positive outcomes at work, this research presents a novel approach by empirically testing a calling conditioned path model from P perception of corporate CSR (P-CSR) to work engagement via meaningfulness under the theoretical framework of self-determination theory. Survey data collected from 224 corporate employees in the US were tested using the PROCESS plugin (version 4.3) in SPSS. The regression results supported the positive direct and indirect paths from employees’ P-CSR to meaningfulness and work engagement but not the conditioning effect of calling work orientation. This study’s unique findings, limitations, future research, and implications are discussed, expanding micro-CSR research and unboxing the management assumptions of employees as purposeful autonomous agents seeking consistent interpretations and authentic perceptions of organizational CSR activities during their sense-making processes. Non-confirming of the calling conditioning the path model shed light on it being a dynamic multi-dimensional and multi-level construct to be further researched.

## 1. Introduction

In a world recovering from the seemingly never-ending global pandemic, researchers have been challenged to solve evolving sustainability crises promptly, including progressive environmental and socio-cultural crises such as global warming, geo-political wars, and economic recessions, while facing polarized public opinions. Simultaneously, some unsettled ongoing research questions for organizations, such as the cost and benefit of corporate social responsibility (CSR), call for timely solutions from individual-level research inquiries on employees’ attitude and psychological reactions toward different business’ CSR practices (i.e., ‘micro-CSR’) [1,2,3]. While over eighty percent of past research focused on inquiries at the institutional level, most CSR researchers still debate whether it is normative or instrumental to the survival of firms as findings on the relationship between CSR and financial performance have been inconclusive [3]. The need for more empirical research on micro-CSR—such as answering the research questions on how employees’ attitudinal and behavioral variables, including perceptions, self-concept, motivation, values, beliefs, job crafting, and career choice, substantiate organizational CSR initiatives through viable individual engagement and well-being—is not just important, it is urgent. This research has the potential to bridge multi-level and multi-disciplinary findings on the impacts of CSR, thereby significantly advancing our understanding of this complex field of sustainability management [2].

## 2. Theoretical Framework

Accumulated micro-CSR research discovered various socio-psychological mechanisms explaining how employees perceive, interpret, react, and participate in organizational CSR activities. These mechanisms, which are the focal constructs of micro-CSR, relate to different employee outcomes [4], such as job satisfaction [5], organizational commitment [6,7,8], citizenship behavior [9,10,11], and turnover intention [12]. Recent meta-analytical research [13] summarized significant existing micro-CSR research evidence. It framed meaningful paths, primarily referring to organizational justice, trust, and identity, under the social exchange theory [14]. The authors [13] called for further research to expand the model, including empirical evidence on employee engagement, creativity, and performance.

Similar to callings from Zhao and colleagues [13] on employee engagement and micro-CSR, several large-scale industry-driven surveys discovered the loss of organizational productivity from employee disengagement [15,16] and called for innovative management solutions. From the self-determination theory (SDT) perspective, when employees are autonomously motivated, they show feelings of energy, empowerment, commitment, and satisfaction [17,18], like the state of being psychologically engaged [19,20]. Cognitive Evaluation Theory (CET) and Organismic Integration Theory (OIT), two of the six mini-theories in the SDT, also emphasize the integrated human experience of self-determined regulation in reaction to external cues to support social functioning and well-being via contributing to the development of others in a community sense and prosocial behavior [21]. This study contributes to micro-CSR research by expending to the SDT as the theoretical framework to empirically examine the relationships among the corporate employees’ perceptions of CSR practices (P-CSR), experienced meaningfulness, and work engagement in the US.

### 2.1. Perceived CSR and Employee Engagement

Work engagement research has shown that one aspect of work engagement, vigor—conceptualized as being full of energy and having the potential of persistent focus, which is an aspect of a eudemonic experience like vitality in the SDT—is particularly beneficial in predicting employee performance and well-being [22]. Moreover, research has shown that when people were autonomously motivated (in a state of highly self-determined and psychologically engaged, [20]), they were more cognitively flexible (open to different organizational cues) [23] and more likely to show a high level of vitality [24]. In the organizational behavior (OB) literature, work engagement [19], often measured by vigor, absorption, and dedication [22], refers to a high level of psychological energy experienced by employees and has been shown to positively predict more adaptive and proactive performance [24] at work. At the same time, adaptivity and proactivity at work also directly indicate employees’ behavioral engagement [19].

To engage at work, employees need to react to different organizational cognitive, linguistic, and conative cues [25] consistently and meaningfully. Cognitive evaluation of daily social functioning at work facilitates or inhibits employees’ sense-seeking thoughts and behavior [4]. P-CSR captures employees’ interpretations of organizational practices and procedures related to CSR philosophies, policies, the effectiveness of senior leadership on CSR, and daily management practices. At the same time, leaders (such as executives and managers) interact with employees regarding these corporate CSR activities at different occasions and frequencies, resulting in different P-CSR. Organizations may keep a regular inventory of CSR activities for statutory or voluntary reporting purposes. Still, authentic and integrated CSR must be a systematic and integrated part of daily operations, reflected in routine business decisions and critical leaders’ attitudes/values. Other than the social exchange theory [14], the SDT [26,27] provides a multi-dimensional motivation framework emphasizing individual employees’ adaptivity to internalize socio-cultural interactions, satisfying their needs for volitional choice (autonomy), impactful outcomes (competence), and meaningful connections (relatedness) [17,28].

Employees’ subjective working experience of their job, referred to as sense-making, activates their motivational and behavioral outcomes [29], such as work engagement and performance. Engaged employees who find organizational culture and senior leadership consistent and supportive in daily management activities are often autonomously motivated [17,27]. In addition, work engagement predicts higher productivity and a higher level of well-being at work [30]. Rather than employees being treated as identical economic agents in the business, with theoretical underlying assumptions that eliminate their choice to craft their jobs according to the social exchange theory [14], we believe employees can be seen as change/serving agents who are organically self-regulating and adapting to achieve the organizational strategic goals (i.e., consistently cued by organizational activities and senior leadership) across different working situations under the SDT [17,26].

Past micro-CSR research has accumulated empirical evidence supporting that employees’ P-CSR positively predicts work outcomes, such as job performance [31] and prosocial behavior [32]. Based on the above reasoning using employee sense-making [33] and the intention to replicate and expand past research on the positive outcomes of perceived CSR through the lens of SDT, the following hypothesis was formed:

**Hypothesis 1.**  *Employees’ P-CSR positively predicts their work engagement*.

### 2.2. Perceived CSR, Work Meaningfulness, and Engagement

Employees’ perceptions of corporate citizenship help align their prosocial values with their employers’ organizational values. Such value alignment builds the foundation for employees’ sense-making [33]. The sense-making process, both at individual and collective levels, can be one of the critical psychological mechanisms for micro-CSR to effectively help select and retain sustainable human capital for long-term and short-term business success [25]. The early proposer of employee engagement, Khan [34], stated that the more employees can bring their true values to their work, the more engagement they will have toward the workplace. Hence, the consistent prosocial values embraced by both organizational practices and employees’ work/life practices formed multi-level unity in shaping the meaningfulness of work [35].

The SDT grounds sense-making processes as an integral part of employees’ self-regulation at work, as employees naturally seek meaningful connections and needs-supportive experiences to grow [17,18,28]. Experiencing the need for a supportive workplace organically mixed with employees’ sense-making at work [4,33]. When organizations invest in authentic and long-term corporate citizenship, contribute to triple bottom lines, and are devoted to the well-being of all stakeholders, they are more likely to attract and retain employees with prosocial values [7]. Consistency and alignment among employees’ P-CSR with responsible management will again reinforce many effective psychological bonds, from supervisors to subordinates, employees to clients, vendors to other colleagues, for efficient strategic collaborations in and outside of the organizations. In this sense, work meaningfulness connects employees’ perceived CSR and work engagement [9]. Hence, the following direct and mediation hypotheses were proposed:

**Hypothesis 2.**  *Employees’ P-CSR positively predicts their work meaningfulness*.

**Hypothesis 3.**  *There is a significant indirect path from employees’ P-CSR to work engagement via work meaningfulness*.

### 2.3. Calling Work Orientation as the Boundary Condition

Work orientation, often distinct among job-career-calling relationships, is a universal subjective attitude people may have toward their work [29]. Employees with calling orientation think of work as inseparable from their lives. They usually see work as socially valuable and involving activities that may not be pleasurable while helping others or achieving higher goals [36]. In this sense, employees’ calling orientation toward their work also facilitates their proactivity into additional but not necessarily directly rewarded activities, such as engaging in recycling requirements and sustainable sourcing practices [37].

Strong subjective perceptions of organizations’ corporate citizenship (i.e., P-CSR) are more compatible with employees high on the calling orientation, who often consider the long-term benefits of organizational corporate citizenship when facing conflict with direct performance-driven incentives. Research also showed a positive relationship between employees’ calling orientation and sense-making at work [38], P-CSR [9], and organizational identity [39]. As a result, employees with high calling work orientation in an organization that emphasizes consistent long-term CSR practices may find their attitudes toward higher-level goals for societal benefits more compatible with their daily on-the-job activities. Hence, the following positive conditioning hypotheses were proposed:

**Hypothesis 4.**  *Calling work orientation positively conditions the relationship between P-CSR and work engagement in the conditioned path model*.

**Hypothesis 5.**  *Calling work orientation positively conditions the relationship between P-CSR and work meaningfulness in the conditioned path model*.

**Figure 1:**
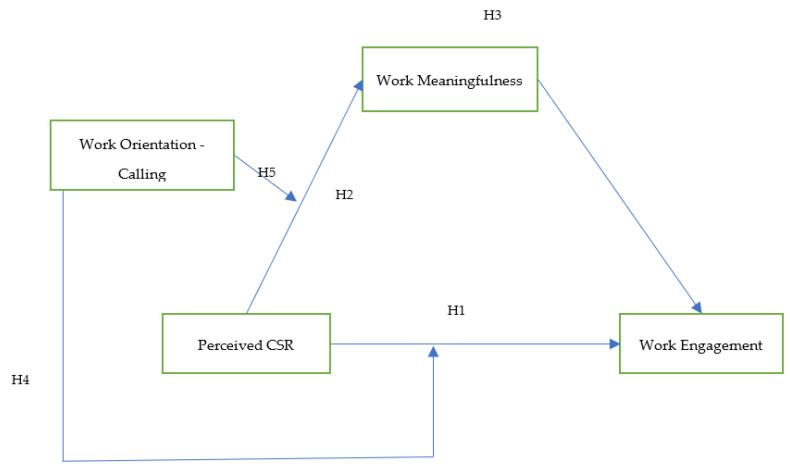
Conditioned path model (moderated mediation model 8, [40]).

Please see Figure 1 as the proposed moderated mediation model for this research.

## 3. Methods

### 3.1. Participants and Procedures

The data collection was conducted in two phases, with the first phase in a private US business before and during the early months of the COVID-19 pandemic, from the fall of 2019 to 2020, which then resumed in 2022/2023 using corporate employee participants recruited via Amazon MTurk. During the two phases, two independent online surveys (T1 and T2 surveys) capturing different variables (independent, and moderate variables in the T1 survey; mediation and dependent variables in the T2 survey) were distributed, with 7–14 days in between them. All participants were notified of the purpose of the survey and their right to withdraw from the survey at any time they wanted. At the end of the T1 survey, participants were reminded that the T2 survey would shortly follow and that their continuance of filling out the following survey would be very important to the research. Employee emails and MTurk worker ID were used to match responses between the T1 and T2 surveys.

A total of 27 employees in the private business were invited to the T1 survey (mean age = 44 years, *SD* = 13 years, mean organizational tenure = 14.85 years; 91% of the participants were male); 10 of these T1-invited employees completed the T2 online survey. There were 537 corporate employees (mean age = 38.63 years, *SD* = 11.91 years, mean organizational tenure = 17.21 years; 60% of the participants were male) recruited from MTurk for the T1 survey; only 214 MTurk participants completed the T2 survey. The average retention ratio for the data collection between T1 and T2 across the different datasets was 38.27% (similar between the employees in the private business and MTurk participants). The final N of the research was 224 participants who completed both the T1 and T2 surveys (mean age = 38.15 years, *SD* = 10.87 years, mean organizational tenure = 16.01 years; 59.55% of the final participants were male).

### 3.2. Measures

The Turker [41] perceived social responsibility scale (*Cronbach’s α* = 0.94) was used to measure employees’ P-CSR. Participants were asked to use a scale of 1 to 7 (with 1 being “strongly disagree” and 7 being “strongly agree”) to assess the statement about the CSR practices of their employer—the organization. There were 18 items (e.g., “Our company contributes to campaigns and projects that promote the well-being of society” and “Our company implements flexible policies to provide a good work and life balance for its employees”) used in this scale to measure the responses.

Work engagement was measured using the short scale developed by Schaufeli and colleagues [22]. Participants were provided with a scale of 1 to 7, with 1 representing “strongly disagree” and 7 representing “strongly agree.” This measurement has 17 statements (Cronbach’s α = 0.93) measuring vigor (6 items; e.g., “At work, I feel full of energy”), absorption (6 items; e.g., “I get carried away when I am working”), and dedication (5 items; e.g., “My job inspires me”).

The indirect path variable of work meaningfulness was measured using the work and meaning inventory by Steger and colleagues [35]. Participants were provided with a scale of 1 to 7, with 1 representing “strongly disagree” and 7 representing “strongly agree.” This measurement has 10 items (Cronbach’s α = 0.92) measuring positive meaning (4 items; e.g., “I have found a meaningful career”), meaning making through work (3 items; e.g., “My work helps me better understand myself”), and greater good motivation (3 items; e.g., “The work I do serves a greater purpose”).

The conditioning variable of work orientation, uniquely the Calling Orientation, was measured using the scales by Bunderson and Thompson [38]. Participants were provided with a scale of 1 to 7, with 1 representing “strongly disagree” and “7” representing “strongly agree.” This measurement has 6 items (Cronbach’s α = 0.94) measuring calling work orientation; example items are “I was meant to be in this line of work” and “My passion for the work I do goes back to my childhood”.

### 3.3. Statistical Analysis

The data were first examined for missing information, and kurtosis and skewness were verified to ensure the univariate normality of the data distribution. Since no significant data were missing, no recompilation of data was conducted. Descriptive statistical analyses and zero-order correlations were then conducted. Finally, an analysis was conducted using the PROCESS (version 4.3) plugin in SPSS to test the proposed conditional path model (moderated mediation; model 8, [40]).

## 4. Results

### 4.1. Descriptive Statistics and Zero-Order Correlations

Please see Table 1 for simple correlations between study variables and the descriptive statistics. All the correlations among the study variables were significant and positive. Skewness and kurtosis test results also demonstrated acceptable normality in this dataset [42].

### 4.2. Conditional Path Analysis—“PROCESS” Results

To test the direct and indirect conditioned path from the perceived CSR to work engagement via work meaningfulness, we used the PROCESS plugin (version 4.3) in SPSS for moderated mediation (model 8, [40]) to examine the proposed conditioning hypotheses. The path model (model 4, [40]) was also run for hypotheses testing to test the direct and indirect path model excluding the moderator.

The PROCESS results showed that P-CSR positively predicted work engagement (*β* = 0.45, *p* < 0.001; see Table 2 and Table 3); hence, hypothesis 1 (H1) about the direct path between perceived CSR and work engagement was supported. P-CSR also positively predicted work meaningfulness (*β* = 0.59, *p* < 0.001; see Table 3), demonstrating that hypothesis 2 (H2) was supported. From testing model 4 [40], the results (*β* = 0.21, *p* < 0.001; see Table 2) showed a significant indirect path from P-CSR to work engagement via work meaningfulness. Therefore, hypothesis 3 (H3) was supported.

The PROCESS results testing the conditioned path model reported no significant moderating effect of calling work orientation. Other than calling work orientation marginally predicting work engagement directly (*β* = 0.32, *p* < 0.10; see Table 4), the interaction term (P-CSR × calling) was not significantly related to either work meaningfulness (*β* = 0.08, *n.s.*; see Table 4) or work engagement (*β* = 0.00, *n.s.*; see Table 4). As a result, hypotheses 4 and 5 (H4 and H5) were not supported.

The conditioned path model indexes were shown in Table 5. Adding the moderating variable of calling work orientation did not improve the path model’s power (i.e., R^2^). The following section discusses the theoretical and practical implications of these findings.

## 5. Discussion

The findings of this research confirmed the positive paths from employees’ P-CSR to work engagement via meaningfulness but not the positive conditioning effects of calling work orientation on these paths. The empirical evidence in this study expanded micro-CSR research and answered management scholars’ calls for more micro-CSR research [2,13]. At the same time, this research enriched the theoretical mechanism of micro-CSR, adding the self-determination theory (SDT) to the repertoire of explanatory mechanisms using theory-driven psychometric measurements [7].

### 5.1. The SDT as a Theoretical Framework for Micro-CSR Research

The direct path between P-CSR and work engagement confirmed that employees’ engagement often aligned with their cognitive appreciation, as part of their sense-making of organizational linguistic, visual, socio-cultural, and operational cues [25]. Daily embedded organizational CSR management can be seen as part of the competitiveness that grows with the organizational human capital. P-CSR directly fuels positive beliefs and attitudes of employees and the significant psychological antecedents of performance and behavioral engagement [15]. In addition, the indirect path from P-CSR to work engagement via meaningfulness also uncovered the limitations on the past common assumption of rewarding employees as the economic agents of the organization, as framed by social exchange theory [14], which provided additional theoretical possibility for sustainability management innovations in organizations under SDT. Furthermore, following the organismic assumptions of human motivation in the SDT [27], employees, as the self-regulated agents working toward the organizational mission and vision, can actively seek out situations and tasks that satisfy their needs for autonomy (e.g., openly and freely pursuing their well-being together with the well-being of others on the job), competence (e.g., understanding individual CO_2_ emission reduction behaviors that are recognized by the company’s values and are seem as part of the collective achievements of the company), and relatedness (e.g., meaningful connections with colleagues/clients/suppliers who share similar socio-cultural values and environmental beliefs). Hence, the more authentic that the employees perceive the organizational CSR practices and leaders’ CSR attitudes/behavior to be, the more meaningful they perceive working for their employer; therefore, they proactively engage, innovatively serve, and actively solve problems at work.

### 5.2. Insignificant Conditioning Results of Calling Work Orientation

Insignificant findings on the positive conditioning effect of calling work orientation (non-confirming of H4 and H5) in this study were fascinating. There has been extensive empirical research on the positive relationship between calling work orientation and the good of life since Wrzesniewski [43] conceptualized people’s subjective work experience into three distinct types: job (i.e., primarily the material benefit from work), career (i.e., achievement and advancement through occupational structure), and calling (i.e., fulfillment through doing the work) orientations. Recent meta-analytical research [44] using studies published in the past two decades found that calling may be hierarchically structured with two correlated, but independent calling types differentiated by their internal and external focuses. Dobrow and colleagues [44] summarized the positive meta-correlations between calling work orientation and work engagement (*r* = 0.49, *p* < 0.001) as well as meaningfulness (*r* = 0.61, *p* < 0.001). However, Dobrow and colleagues [44] categorized the measurement of calling [38], the one used in this study, as the measurement with internal focus (see Table 1, [44] p. 516), which relates to subjective well-being via self-realization/job satisfaction in their newly proposed theoretical framework. In addition, they theorized that an externally focused calling is characterized by doing work that addresses the needs of society and paths to broadened psychological well-being via meaningful work. This meta-analytical paper [44] provided a possible explanation for the non-confirming findings in this study, such as the use of incompatible measurements. Future empirical research is needed to further validate Dobrow and colleagues’ theoretical framework.

Another recent theoretical study of work orientation conducted by Schabram and colleagues [45] reframed the three mutually exclusive types of work orientation, including job, career, and calling, into four types of work orientation profiles using a two-dimensional (calling by career) model. Schabram and colleagues [45] not only progressed the theoretical framework of work orientation but also integrated empirical analysis of research evidence regarding the temporal dynamics and latent profiles in different types of work orientation. Their findings also suggested that multiple types/profiles of work orientations must be measured and studied simultaneously with a sophisticated longitudinal research design.

## 6. Limitations

One limitation of this study is that some research participants were from a private corporation (about 10%) while others (about 90% of the final sample) were recruited via Amazon MTurk. Hence, our sample may include unexplained heterogeneity other than in the variations of the intended measurements. Another limitation of this research was that the time of data collection was separated by years (i.e., before and after the COVID-19 pandemic). Since the study only used a limited number of participants before the onset of COVID-19, no significant impact on the findings was anticipated. Lastly, the research design was cross-sectional and we cannot guarantee the determination of causal relationships among the primary constructs in the study’s general research model. Hence, caution is needed when using the research findings of this study to explain practical issues that may arise in organizations.

## 7. Conclusions

The study contributes to the understanding of micro-CSR and its impact on employee outcomes [2], emphasizing the role of P-CSR in shaping employees’ sense of meaningfulness and engagement at work under the theoretical framework of the SDT [26]. The non-significant conditioning effects of calling work orientation suggest further exploring meaningful individual differences for sustainability management and their interactions with various work contexts. With a humanistic approach emphasizing alignment between employees’ and organizational values and belief in sustainability [13], business practices that support employees’ needs for autonomy, relatedness, and competence can be seen as bridging organizational CSR initiatives to employees’ sense-making, self-regulation, and engaged work behavior. Overall, this study sheds light on the intricate relationships between P-CSR, work meaningfulness, and engagement, offering insights for organizations seeking to enhance employee well-being and performance through authentic and effective CSR initiatives [13,26]. Such efforts may eventually result in higher customer satisfaction and sustainable community contributions. Further research exploring meaningful individual differences in sustainability management and its longitudinal effects is warranted to advance our understanding of micro-CSR dynamics in organizational contexts.

## Figures and Tables

**Table 1:** Means, standard deviations, skewness, kurtosis, and correlations summary.

	1	2	3	4	Mean	SD	Skewness		Kurtosis	
							*Statistic*	*Std. err*	*Statistic*	*Std. err*
**1. P-CSR**	-				5.22	1.11	1.09	0.16	1.13	0.32
**2. Work Orientation—Calling**	0.61 **	-			4.89	1.56	1.13	0.16	0.37	0.32
**3. Work Meaningfulness**	0.58 **	0.63 **	-		5.25	1.13	1.20	0.16	1.68	0.32
**4. Work Engagement**	0.71 **	0.76 **	0.66 **	-	5.15	1.04	1.08	0.16	1.65	0.32

*Note.* N = 224; *SD* = standard deviation; ** *p* < 0.001.

**Table 2:** Coefficients for direct and indirect effects of P-CSR on work engagement via work meaningfulness.

	Direct/Indirect Effects						
**IV**		*X* *->* *Y*	*95% CI*		*(X* *->* *M* *->* *Y)*	*95% CI*	
**P-CSR (model 4)**		0.45 **	[0.28	0.62]	0.21 **	[0.10	0.35]
**P-CSR (model 8)**		X -> Y	95% CI		(X -> M -> Y)	95% CI	
	*W Mean*	0.30 **	[0.13	0.48]	0.04 **	[0.00	0.13]
	*W Mean + 1SD*	0.30 **	[0.18	0.41]	0.07 **	[0.02	0.13]
	*W Mean + 2SD*	0.29 **	[0.16	0.42]	0.09 **	[0.03	0.16]

*Note*. N = 222 (list-wise); PCSR = perceived corporate social responsibility; IV = independent variable; CI = confidence interval; ** *p* < 0.001. X refers to the independent variables (IV; perceived corporate social responsibilities), M refers to the mediator (work meaningfulness), W refers to the moderator (calling work orientation), and Y refers to the dependent variable (work engagement). In model 4 [40], the indirect effect of X on Y through Mi = *a_i_b_i_* and the direct effect of X on Y = *c*_1_’. In model 8 [40], the conditional indirect effect of X on Y through Mi = (*a_i_* + *a_3i_*W)*b_i_* and the conditional direct effect of X on Y = *c*_1_’ + *c*_3_’ W; both conditional direct and indirect effects were reported with different levels of W.

**Table 3:** Path model parameter summary (without the moderator).

	Work Meaningfulness (Mediator; M_i_)			Work Engagement (DV; Y)		
	*Coeffe*	*BootMean*	*BootSE*	*Coeffe*	*BootMean*	*BootSE*
**P-CSR (IV, X) - *a_i_***	0.59 **	0.59	0.08			
**WM (M_i_) - *b_i_***				0.35 **	0.36	0.09
**P-CSR (X) - *c’***				0.45 **	0.44	0.08

*Note.* P-CSR = perceived corporate social responsibility; calling = calling work orientation; WM = work meaningfulness; IV = independent variable; DV = dependent variable; coeffe = coefficient; N = 222 (list-wise); ** *p* < 0.001. X refers to the independent variables (IV; perceived corporate social responsibilities), M refers to the mediator (work meaningfulness), and Y refers to the dependent variable (work engagement). In model 4 [40], the indirect effect of X on Y through Mi = *a_i_b_i_* and the direct effect of X on Y = *c*_1_’; coefficients and standardized error after bootstrapping (i.e., 5000) were reported as BootMean and BootSE.

**Table 4:** Conditioned path analyses parameters summary.

	Work Meaningfulness (Mediator; M_i_)			Work Engagement (DV; Y)		
	*Coeffe*	*BootMean*	*BootSE*	*Coeffe*	*BootMean*	*BootSE*
**P-CSR (IV, X) - *a*_1*i*_**	−0.05	−0.02	0.29			
**Calling (W) - *a*_2*i*_**	−0.08	−0.06	0.27			
**P-CSR** × **Calling (XW) - *a*_3*i*_**	0.08	0.08	0.05			
**WM (M_i_) - *b_i_***				0.19 *	0.20	0.07
**P-CSR (X) - *c*_1_’**				0.32 †	0.28	0.19
**Calling (W) - *c*_2_’**				0.31 †	0.28	0.18
**P-CSR** × **Calling (XW) -*c*_3_’**				0.00	0.00	0.03

*Note.* P-CSR = perceived corporate social responsibility; calling = calling work orientation; WM = work meaningfulness; IV = independent variable; DV = dependent variable; coeffe = coefficient; N = 222 (list-wise deletion); * *p* < 0.05; † *p* < 0.10. X refers to the independent variables (IV; perceived corporate social responsibilities), M refers to the mediator (work meaningfulness), W refers to the moderator (calling work orientation), and Y refers to the dependent variable (work engagement). In model 8 [40], the conditional indirect effect of X on Y through Mi = (*a_i_* + *a*_3*i*_ W)*b_i_* and the conditional direct effect of X on Y = *c*_1_’ + *c*_3_’ W; coefficients and standardized error after bootstrapping (i.e., 5000) were reported as BootMean and BootSE.

**Table 5:** Conditioned path model indexes.

	Work Meaningfulness (M_i_)			Work Engagement (DV)		
	R^2^	F	sig.	R^2^	F	sig.
Model 8	0.47	54.43	0.00	0.59	90.67	0.00
Model 4	0.58	48.62	0.00	0.59	108.7	0.00

*Note.* Model 8 was the conditioned path model with perceived corporate social responsibilities as the independent variable, work meaningfulness as the mediator, calling work orientation as the moderator, and work engagement as the dependent variable. Model 4 was the path model with perceived corporate social responsibility as the independent variable, work meaningfulness as the mediator, and work engagement as the dependent variable.

## Data Availability

The original contributions presented in the study are included in the article, further inquiries can be directed to the corresponding author.
